# Neurological risks of COVID-19 in women: the complex immunology underpinning sex differences

**DOI:** 10.3389/fimmu.2023.1281310

**Published:** 2023-11-14

**Authors:** Jienan Gu, Jiale Zhang, Qianhui Liu, Shijie Xu

**Affiliations:** ^1^ Institute of Basic Theory for Chinese Medicine, China Academy of Chinese Medical Sciences, Beijing, China; ^2^ The First School of Clinical Medicine, Zhejiang Chinese Medical University, Hangzhou, China

**Keywords:** COVID-19, women, autoimmunity, neuroinflammation, X-chromosome

## Abstract

The COVID-19 pandemic has uncovered many mysteries about SARS-CoV-2, including its potential to trigger abnormal autoimmune responses. Emerging evidence suggests women may face higher risks from COVID-induced autoimmunity manifesting as persistent neurological symptoms. Elucidating the mechanisms underlying this female susceptibility is now imperative. We synthesize key insights from existing studies on how COVID-19 infection can lead to immune tolerance loss, enabling autoreactive antibodies and lymphocyte production. These antibodies and lymphocytes infiltrate the central nervous system. Female sex hormones like estrogen and X-chromosome mediated effects likely contribute to dysregulated humoral immunity and cytokine profiles among women, increasing their predisposition. COVID-19 may also disrupt the delicate immunological balance of the female microbiome. These perturbations precipitate damage to neural damage through mechanisms like demyelination, neuroinflammation, and neurodegeneration – consistent with the observed neurological sequelae in women. An intentional focus on elucidating sex differences in COVID-19 pathogenesis is now needed to inform prognosis assessments and tailored interventions for female patients. From clinical monitoring to evaluating emerging immunomodulatory therapies, a nuanced women-centered approach considering the hormonal status and immunobiology will be vital to ensure equitable outcomes. Overall, deeper insights into the apparent female specificity of COVID-induced autoimmunity will accelerate the development of solutions mitigating associated neurological harm.

## 1 Introduction

The COVID-19 pandemic, caused by the novel coronavirus SARS-CoV-2 has been associated with autoimmune responses in some patients. Autoimmunity arises when the immune system loses tolerance to self-antigens and produces autoantibodies attacking host tissues (1). Both cellular and humoral autoimmune reactions have been described in COVID-19 patients (2, 3). For example, anti-interferon autoantibodies capable of impairing antiviral responses are detected in approximately 10.2% of severe COVID-19 cases (4). Additionally, SARS-CoV-2 binding to tissue antigens may induce cross-reactivity of immune cells and subsequent autoimmune damage in organs like the Livers and nervous system (5–7). Understanding the autoimmune aspects of COVID-19 is crucial, as they may exacerbate disease severity and cause prolonged symptoms in recovered patients.

Emerging evidence suggests that COVID-19 can trigger autoimmune responses through several mechanisms. Viral infections often provoke autoimmunity via molecular mimicry, wherein viral antigens resemble self-antigens (8). SARS-CoV-2 proteins may share sequences or structures with host proteins, leading to cross-reactivity of antibodies or T cells (5). Additionally, the severe inflammation and cytokine storm induced by COVID-19 may cause the breakdown of self-tolerance. Elevated levels of cytokines like IL-6, IL-17, and TNF-α can stimulate auto-reactive lymphocytes (9–11). SARS-CoV-2 infection can also prompt neutrophil extracellular trap (NET) formation and release of danger-associated molecular patterns (DAMPs), further enhancing immune dysregulation (12, 13). Identifying the pathways leading to autoimmunity following COVID-19 is imperative to improve understanding and management of the disease.

Autoimmune responses have been shown to impact and damage both the central and peripheral nervous systems. Autoimmune diseases like multiple sclerosis, Guillain-Barre syndrome, involve neural inflammation, demyelination, and neurodegeneration (14, 15). Autoantibodies can also directly attack neurons and synaptic connections, disrupting neural signaling (16). Moreover, increasing evidence indicates that acute infections can also elicit autoantibodies to cause cross-reactivity against the nervous system (17). Therefore, the autoimmune reactions associated with COVID-19 infection may similarly lead to neurological damage. Elucidating how autoimmunity induced by SARS-CoV-2 affects the nervous system is critical for managing neurological sequelae.

COVID-19 may disproportionately impact the female nervous system. While there is no evidence that women infected with SARS-CoV-2 have higher rates of acute neurological complications like stroke, female sex has been identified as an independent risk factor for developing long COVID syndromes (18, 19). Additionally, neuropsychiatric symptoms such as anxiety and depression are commonly reported neurological manifestations of long COVID, with a higher proportion of women experiencing these symptoms compared to men (19). The mechanisms underlying this female propensity for neuro-COVID sequelae are still unclear. However, sex differences in immune responses and hormonal influences may contribute to increased susceptibility of the female nervous system to long-term damage mediated by COVID-19. Moreover, autoimmune diseases like multiple sclerosis and rheumatoid arthritis that affect the nervous system are more prevalent in women (20–22).

## 2 Autoimmunity and nervous system

Contemporary research has uncovered dynamic interplays between the central nervous system (CNS) and the immune system, challenging the erstwhile notion of CNS immune privilege (23). Specific niches such as the choroid plexus, meninges, and perivascular spaces, along with the meningeal lymphatic system and skull microchannels, facilitate ongoing communication between the brain and the immune system, essential for CNS maintenance, function, and repair (23, 24). The blood-brain barrier (BBB), constituted by tightly connected brain endothelial cells, still plays a crucial role in limiting the entry of pathogens and activated immune cells into the CNS, thereby protecting neurons (25). In parallel, intrinsic brain cells like microglial cells and astrocytes monitor pathogen invasion and tissue damage, initiating moderate neuroinflammatory responses when necessary (26). This nuanced immune interaction forms a balanced immune environment essential for maintaining CNS homeostasis (27). However, infections have the potential to disrupt the balanced immune interaction by introducing inflammatory factors from outside the CNS, which might alter the permeability of the BBB, potentially allowing peripheral immune cells to enter the CNS (28, 29). The influx of inflammatory cells and cytokines into brain tissue can activate glial cells and trigger neuroinflammation (28). This may lead to autoantigen exposure and loss of immune tolerance, which in turn produces autoreactive T cells and antibodies that attack neural tissues. Particularly, the interaction between SARS-CoV-2 and the CNS could exacerbate these responses, potentially leading to severe neurological complications.

### 2.1 Mechanisms of autoimmunity against the nervous system

Molecular mimicry, a well-documented mechanism for infection-induced autoimmunity, arises from amino acid sequence or structural similarities between pathogen components and host proteins, leading to cross-reactivity of antibodies and T cells (8). This has been demonstrated for several neurotropic viruses, wherein immune cells primed by the virus later erroneously recognize and attack similar epitopes on nervous system antigens (30, 31). Analogously, molecular mimicry between SARS-CoV-2 and neuronal proteins is hypothesized to facilitate COVID-19 associated autoimmune neuropathology. The S1 protein of the SARS-CoV-2 spike has been found to share sequence homology with a number of CNS proteins, though cross-reactivity remains to be confirmed experimentally (32).

The severe inflammation and cytokine storm associated with severe COVID-19 may also promote autoimmune reactivity against the nervous system. Elevated levels of pro-inflammatory cytokines like IFN-γ, TNF-α, IL-6 and IL-17 can activate self-reactive T cells and B cells that have escaped tolerance mechanisms (33, 34). Numerous neurologic autoimmune diseases demonstrate associations with such pro-inflammatory cytokines. For instance, IFN-γ and IL-17 have been implicated in multiple sclerosis, with therapeutic blockade of these cytokines conferring benefits (35–37). Dampening the excessive inflammation in critical COVID-19 cases may help mitigate collateral autoimmune damage to the nervous system.

Cross-reactivity of immune cells primed by SARS-CoV-2 with CNS antigens provides another avenue for COVID-19 associated autoimmunity. The virus binding to ACE2 receptors on the surface of nervous system cells may prompt the formation of anti-SARS-CoV-2 antibodies or T cells capable of recognizing similar host cell surface features (32). Additionally, damage and release of sequestered CNS proteins due to viral infection can expose neo-epitopes and trigger new autoreactive clones (8, 38). For instance, immune responses targeting the virus nucleocapsid protein were found to also cross-react with host small nuclear ribonucleoprotein particles in the brain (39, 40). Identifying such potentially cross-reactive immune targets would enable a more accurate evaluation of autoimmune risk following COVID-19.

### 2.2 Nervous system dysfunction caused by autoimmunity

Autoimmune reactions can trigger neuroinflammation that impairs nervous system function. Infiltration of autoreactive T cells and autoantibodies activating microglia can establish chronic inflammatory foci in the brain and spinal cord (41). Enhanced levels of inflammatory cytokines disrupt neuronal signaling and alter neurotransmitter levels, while also weakening the blood-brain barrier (42, 43). Similar neuroinflammation is thought to underlie some neurological symptoms of long COVID, as autoimmunity triggered by SARS-CoV-2 persists even after viral clearance (44). Imaging studies in these patients have revealed microstructural changes in brain regions that regulate emotion, memory and cognition - aligning with symptoms like brain fog.

Demyelination due to autoimmune targeting of myelin sheaths is another major mechanism of nervous system damage. Destruction of myelin insulation around axons by autoantibodies and autoreactive lymphocytes leads to slow nerve conduction and neurological deficits (45, 46). Demyelinating diseases like multiple sclerosis and acute disseminated encephalomyelitis often follow viral or bacterial infections, via mechanisms like molecular mimicry (47). Although demyelination has not yet been specifically examined in neuro-COVID, it represents a plausible pathological consequence of COVID-19 induced autoimmunity. Demyelinating autoantibodies or T cells arising from cross-reactivity with SARS-CoV-2 components could manifest in neurological sequelae like fatigue, numbness and nerve pain.

Autoimmune-mediated neurodegeneration is characterized by progressive loss of neurons and neural networks. The binding of autoreactive antibodies to neurons, prion-like misfolding of proteins triggered by autoimmunity, and indirect damage via inflammation can all promote neurodegeneration (48, 49). Viral infections are often considered triggers for such pathology, as seen in HIV-associated dementia and post-encephalitic Parkinsonism (50, 51). Though not yet conclusively demonstrated, the long-term persistence of inflammation and autoimmunity associated with COVID-19 raises concerns about the risk of insidious neurodegenerative changes. Monitoring biomarkers and imaging indicators of neurodegeneration will be important among recovered COVID-19 patients, especially those reporting neurological complaints.

### 2.3 Glial cell and vascular damage induced by autoimmunity

Autoimmune responses triggered by COVID-19 may also contribute to nervous system injury by targeting glial cells and blood vessels. Autoantibodies binding astrocytes and oligodendrocytes can disrupt the homeostasis and function of these glial cells, which support and interact with neurons (52). Anti-endothelial cell autoantibodies can promote apoptosis of vascular endothelial cells lining the blood-brain barrier, leading to microhemorrhages and facilitating neuroinflammation, and although this increase is uncommon in infected patients in the acute phase of COVID-19, it cannot be ruled out in long COVID (53). A study indicates patients with COVID-19 associated encephalopathy were found to have autoantibodies binding components in the brain, though specific targets remain unclear (54). Further research is still needed to confirm and characterize autoimmune mediated damage to glial cells, neurons and the vasculature in SARS-CoV-2 infection. Understanding the mechanisms of COVID-19 elicited autoimmunity warrants urgent investigation to guide the management of neurological sequelae.

## 3 COVID-19, autoimmunity and female specificity

### 3.1 Sex differences in COVID-19-induced autoimmunity

Emerging evidence indicates that women exhibit distinct autoimmune responses following COVID-19 compared to men. Female COVID-19 patients were found to have higher frequencies of various autoantibodies like antiphospholipid antibodies (55). Furthermore, women tend to have more robust antibody reactions to the SARS-CoV-2 spike protein antigen compared to males (21, 56). These early findings suggest that female-specific factors may promote augmented autoimmunity following COVID-19 infection. Understanding the mechanisms underlying this sex bias could have implications for the diagnosis, monitoring, and management of neurological sequelae that may persist longer in women.

### 3.2 Possible mechanisms of female specificity

Sex hormones like estrogen, progesterone and testosterone can modulate immune responses and may contribute to the heightened autoimmunity seen in female COVID-19 patients. Estrogen tends to promote more vigorous humoral immunity and antibody production compared to androgens like testosterone (57). The cyclic fluctuations in estrogen and progesterone levels over the menstrual cycle also drive periodic changes in immune activity (58). Therefore, hormonal differences in females may create a pro-inflammatory cytokine milieu and enhanced B cell responses conducive to developing autoreactive antibodies after viral infections like COVID-19 (21, 22). Further research is required to delineate the complex interplay between sex hormones and autoimmunity following SARS-CoV-2 infection.

**Figure 1:**
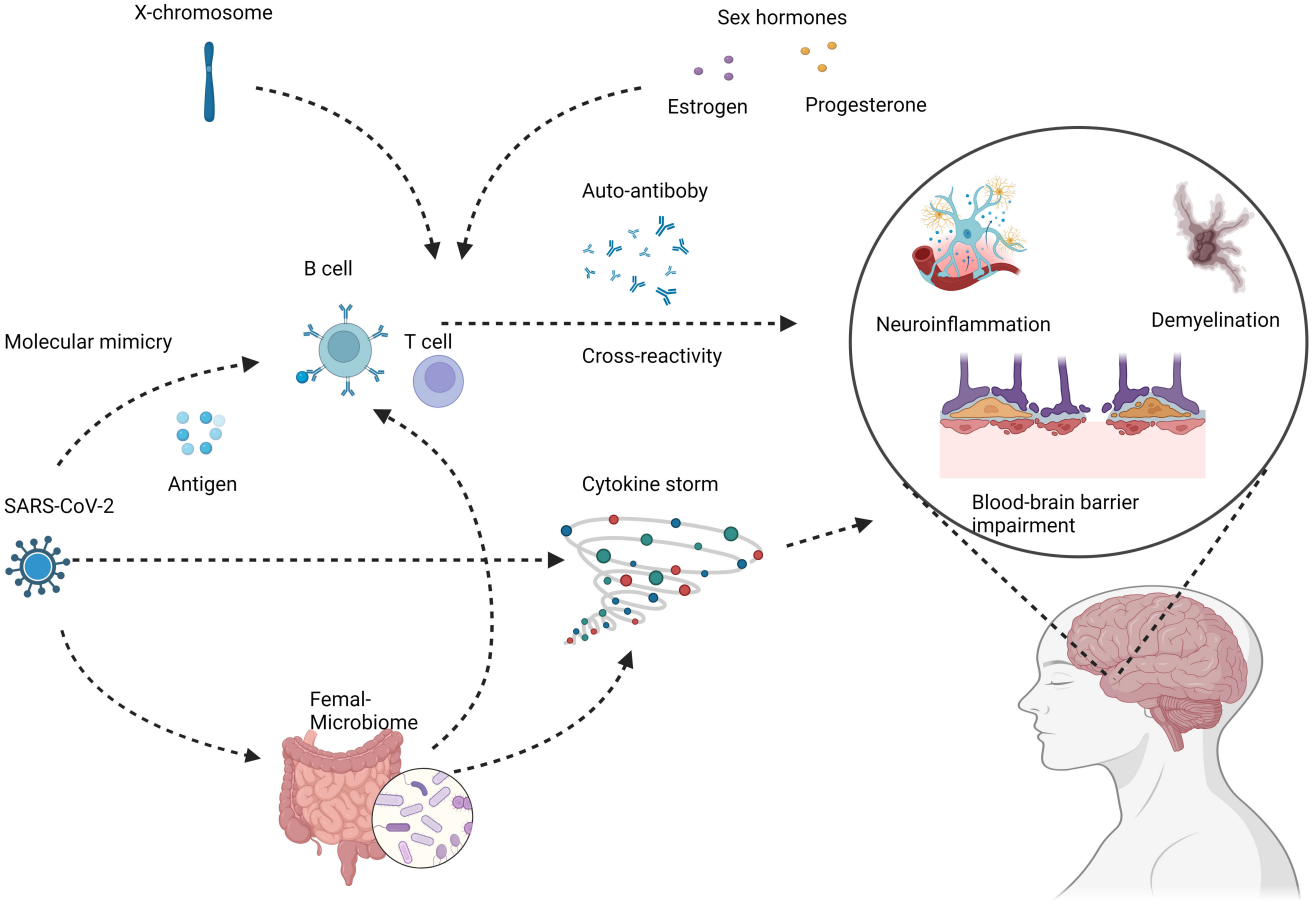
Potential mechanisms underlying female-specific risks of COVID-19 induced autoimmunity. COVID-19 infection can lead to loss of immune tolerance and production of autoreactive immune cells and antibodies through mechanisms like molecular mimicry and cytokine storm. These aberrant immune responses can target components of the nervous system, including neurons, glial cells, and the blood-brain barrier. The subsequent autoimmune attack manifests as neuroinflammation, demyelination, and neurodegeneration, potentially increasing the risks of chronic neurologic conditions like multiple sclerosis. Female COVID-19 patients demonstrate distinct immune dysregulation features such as higher autoantibody levels, skewed cytokine profiles, and enhanced B cell reactivity. Contributing factors likely involve sex hormones, X chromosome effects, and microbiome alterations. These specifically predispose women to COVID-induced autoimmunity that damages the nervous system. Created with BioRender.com.

The increased copy number of X chromosomes in females has also been implicated in the sexual dimorphism of autoimmunity. Several immune-related genes are located on the X chromosome, including TLR7 involved in antiviral responses (59–61). Higher expression of such genes in females due to X-chromosome mosaicism may promote stronger inflammatory reactions to viruses like SARS-CoV-2 (62, 63). Additionally, X chromosome inactivation is a complex process that generally helps achieve dosage compensation between XX females and XY males (64, 65). However, this process is imperfect and can lead to minor differences in gene expression between sexes (66). These subtle expression differences likely contribute to increased autoimmunity in females (66), the potential mechanisms underlying female-specific risks of COVID-19 induced autoimmunity show in **Figure 1**. These X-linked effects likely interact with hormonal influences to create a female-specific risk profile for developing autoantibodies and dysregulated immunity after COVID-19 infection.

Differences in the gut and reproductive tract microbiota between males and females could also help explain the increased COVID-19 associated autoimmunity in women. The female microbiome exhibits a greater abundance of certain bacteria that shape immune function (67). Dysbiosis of the female microbiota is also associated with higher risks of autoimmunity (68). For instance, bacterial genera such as *Alistipes*, *Akkermansia*, *Eggerthella*, *Blautia*, *Pseudoflavonifractor*, *Anaerotruncus*, and *Clostridium*, among others, are found to be more prevalent in females (69). Specifically, *Akkermansia muciniphila* and *Eggerthella lenta* have been implicated in the modulation of immune responses, with the former potentially having negative effects on certain neurological/autoimmune diseases like Multiple Sclerosis (70), and the latter promoting Th17 cell activation, thus exacerbating colitis and being enriched in various autoimmune diseases including Inflammatory Bowel Disease (IBD) (71, 72). Similarly, *Anaerotruncus colihominis* has been associated with the severity of Experimental Autoimmune Encephalomyelitis (EAE) in mice, a model for Multiple Sclerosis (73). The perturbation of the delicate balance of microflora by SARS-CoV-2 infection in susceptible individuals (74, 75), could further exacerbate this scenario, potentially leading to heightened autoimmune pathology preferentially in females (74, 76). Further characterization of the sexual dimorphism in microbiota composition and function could elucidate the role of the microbiome in the sex-biased autoimmune outcomes of COVID-19 observed clinically.

### 3.3 Observed neurological symptoms in COVID-19 female patients

A recent prospective cohort study of patients with mild COVID-19 found that female patients reported more neurologic and neuropsychiatric symptoms, like cognitive deficits, headaches, and hyposmia compared to males (77). However, the study did not compare COVID-19 patients to a control group without COVID-19, thus it remains unclear whether the observed sex differences represent increased neuro-autoimmunity, specifically caused by COVID-19 in women (77). Several factors like autoantibody-mediated microvascular damage may underlie the sex differences in neurological manifestations of COVID-19 (78), a retrospective study also found female patients had a higher frequency of certain neurological post-COVID symptoms, though mechanisms need further study (79). More research is still needed to elucidate the pathological mechanisms and determine if female sex is truly a risk factor for neurological sequelae after COVID-19 infection (77).

## 4 Perspective and future directions

The apparent female predisposition for neurological complications and autoimmunity following COVID-19 highlights the need to incorporate sex-based analyses into ongoing and future studies. All aspects of COVID-19 research should include female-specific cohorts to delineate differences in disease course, pathogenesis, and outcomes between the sexes (80). Mechanistic studies should aim to uncover the immunological, hormonal, genetic, and microbial factors driving the distinct female neuro-COVID manifestations (21). Such research efforts will enable more precise clinical monitoring, prognostication, and management tailored to female patients during acute infection and through long COVID (81). Overall, intentionally embracing female inclusivity and sex-comparisons in the basic, translational and clinical science of COVID-19 will ensure equitable biomedical progress for women.

The sex-specific characteristics of COVID-19 autoimmunity and neurological sequelae warrant the development of tailored therapeutic strategies for women. Hormonal modulation to stabilize immune dysregulation in female patients represents one approach (82). Personalized immunomodulators based on a woman’s menstrual cycle stage or menopause status may also prove beneficial (83, 84). Additionally, gut microbiome modification and pre/probiotics could help counteract any COVID-19 induced dysbiosis known to enable autoimmunity, which appears to preferentially affect the female microbiota (85, 86). Repurposing approved treatments for autoimmune illnesses with female predominance may provide faster solutions. Ultimately, any immunomodulatory or neuroprotective approaches to managing long COVID should consider female-specific metrics and mechanisms given the apparent sexual dimorphism of the underlying pathology.

The care of female COVID-19 patients should account for their potentially heightened risk of autoimmune-mediated neurological sequelae (87). Clinicians should maintain a high index of suspicion for neuropsychiatric symptoms and autoimmunity in women after COVID-19 (88). Extended monitoring for signs of neuroinflammation, subclinical autoantibodies and thrombotic markers may enable earlier intervention (89). Telemedicine can enhance access to appropriate care while remote patient-reported tracking of symptoms enables personalized evaluation. Overall, a nuanced clinical approach conscious of sex differences will be key to improving long-term outcomes in female COVID-19 survivors.

## 5 Conclusion

In summary, COVID-19 can elicit autoimmune responses that appear to disproportionately affect the female nervous system. The infection triggers elevated pro-inflammatory cytokines, autoreactive lymphocytes, and autoantibodies that can bind to or cross-react with neural antigens (17). Elevated cytokines, autoreactive immune cells, and autoantibodies triggered by the infection could potentially bind neurons and glial cells, leading to damage like neuroinflammation and demyelination (90, 91). Female-specific factors such as hormones, X chromosome effects, and microbiota composition likely interact to heighten COVID-induced autoimmunity (22). Elucidating the sex differences in COVID-19 pathogenesis and neurological outcomes remains an urgent priority. Characterizing the responsible mechanisms will pave the way for potential immunomodulatory treatments and female-centered clinical monitoring to improve long-term neural health after COVID-19.

COVID-19 can trigger autoimmune responses that damage the nervous system (92). Some early studies have reported more neurological symptoms in female COVID-19 patients compared to males (93–95). Mechanisms like molecular mimicry and cytokine storm may enable COVID-autoimmunity (32). Female-specific factors including hormones and X-chromosome effects likely contribute (22). Understanding female-specific risks is vital to guide monitoring and therapies for neuro-COVID (96). Overall, the perspective emerging from early COVID-19 studies indicates that the interplay between SARS-CoV-2 infection and autoimmunity may pose a particularly insidious threat to the female nervous system. Further research intentionally examining female specificity in pathogenesis and outcomes will be crucial going forward.

Looking ahead, further investigation of sex-specific neurological effects of COVID-19 is imperative. Longitudinal studies tracking female patients will uncover the longer-term autoimmune impacts of the illness. Mechanistic research should delineate the contributions of hormonal fluctuations, X chromosome effects and immunological factors underlying the female predisposition (22). Multidisciplinary collaborations between neuroscientists, immunologists and clinicians will be key to unraveling the complex pathogenesis (97). Ultimately, insights from sex-based analyses can inform prognostic models to identify women at heightened risk of neurologic sequelae post-COVID. They may also guide clinical decision-making on the optimal timing and choice of immunomodulatory interventions to mitigate chronic neurologic damage in the expanding population of female COVID-19 survivors. An intentional focus on addressing female-specific risks will accelerate progress in improving the lives of women affected by this devastating illness.

## Data availability statement

The raw data supporting the conclusions of this article will be made available by the authors, without undue reservation.

## Author contributions

JG: Writing – original draft, Writing – review & editing. JZ: Writing – original draft, Writing – review & editing. QL: Writing – original draft, Writing – review & editing. SX: Writing – original draft, Writing – review & editing.
